# Supplementary material for: MetaRibo-Seq measures translation in microbiomes
Source: Nat Commun. 2020 Jun 29;11:3268. doi: 10.1038/s41467-020-17081-z (PMC7324362; doi:10.1038/s41467-020-17081-z)
Supplement: Supplementary file 10 — Supplementary Data 7 [file 41467_2020_17081_MOESM10_ESM.zip › File2/Confidence_High_Taxonomy/305733_out.krona.html]

Javascript must be enabled to view this page.

members
magnitude
magnitudeUnassigned
count
unassigned
taxon
rank

305733\_out

116

116
superkingdom
2

976
phylum
116

class
116
200643

116
order
171549

family
3
1853231

2
genus
574697


SRS145497\_contig\_number\_13548SRS148196\_contig\_number\_37457
species
2
1472416

genus
1
283168

1
species

SRS018888\_contig\_number\_1941
28118

2005525
family
105

375288
genus
1
105

SRS013158\_contig\_number\_3131

species

SRS019496\_contig\_number\_12369SRS1041129\_contig\_number\_7027
2
1262912

1

SRS015794\_contig\_number\_3049
species
2293116

1
species

SRS023971\_contig\_number\_4248
658662

563193

SRS016335\_contig\_number\_45062SRS056273\_contig\_number\_32891SRS075021\_contig\_number\_7306
species
3

823
95
species

SRS011134\_contig\_number\_49633SRS011239\_contig\_number\_11898SRS013098\_contig\_number\_35218SRS013638\_contig\_number\_7937SRS013800\_contig\_number\_22373SRS014235\_contig\_number\_32528SRS014313\_contig\_number\_1213SRS014412\_contig\_number\_12867SRS014459\_contig\_number\_21149SRS015065\_contig\_number\_14624SRS015217\_contig\_number\_contig-100\_2427.165098SRS016056\_contig\_number\_663SRS016095\_contig\_number\_11659SRS016267\_contig\_number\_10164SRS017521\_contig\_number\_38948SRS018541\_contig\_number\_10230SRS018656\_contig\_number\_12053SRS018836\_contig\_number\_14511SRS018984\_contig\_number\_25569SRS019178\_contig\_number\_1826SRS019267\_contig\_number\_contig-100\_726.58191SRS019397\_contig\_number\_3875SRS019910\_contig\_number\_24097SRS020394\_contig\_number\_14628SRS022609\_contig\_number\_48697SRS023583\_contig\_number\_16807SRS023829\_contig\_number\_contig-100\_1339.1339SRS043701\_contig\_number\_10116SRS043768\_contig\_number\_24114SRS045195\_contig\_number\_6523SRS045713\_contig\_number\_21420SRS045826\_contig\_number\_19651SRS048060\_contig\_number\_10622SRS049712\_contig\_number\_contig-100\_2681.120123SRS049773\_contig\_number\_17505SRS049900\_contig\_number\_19158SRS051610\_contig\_number\_1545SRS052027\_contig\_number\_12907SRS052078\_contig\_number\_1473SRS053398\_contig\_number\_contig-100\_443.158984SRS053649\_contig\_number\_7827SRS054590\_contig\_number\_40SRS056259\_contig\_number\_8670SRS058145\_contig\_number\_2440SRS058723\_contig\_number\_6832SRS062701\_contig\_number\_1899SRS063040\_contig\_number\_44735SRS064557\_contig\_number\_16995SRS064645\_contig\_number\_1263SRS065397\_contig\_number\_13170SRS065504\_contig\_number\_contig-100\_719.186539SRS076804\_contig\_number\_11386SRS077127\_contig\_number\_9642SRS077194\_contig\_number\_12817SRS077589\_contig\_number\_4372SRS078176\_contig\_number\_1637SRS078242\_contig\_number\_20461SRS078419\_contig\_number\_28972SRS097920\_contig\_number\_19412SRS097958\_contig\_number\_11451SRS098061\_contig\_number\_7432SRS098655\_contig\_number\_2443SRS100021\_contig\_number\_423SRS104036\_contig\_number\_6454SRS1041032\_contig\_number\_1088SRS1041036\_contig\_number\_805SRS1041092\_contig\_number\_8908SRS1041138\_contig\_number\_4372SRS1041142\_contig\_number\_675SRS104311\_contig\_number\_5106SRS104327\_contig\_number\_863SRS1055038\_contig\_number\_810SRS1055049\_contig\_number\_10699SRS1055095\_contig\_number\_contig-100\_1308.1309SRS140492\_contig\_number\_33908SRS142542\_contig\_number\_26298SRS142712\_contig\_number\_1509SRS143148\_contig\_number\_2685SRS143342\_contig\_number\_2113SRS143523\_contig\_number\_2109SRS143876\_contig\_number\_15445SRS144297\_contig\_number\_13705SRS144506\_contig\_number\_4276SRS146813\_contig\_number\_2956SRS147272\_contig\_number\_505SRS147614\_contig\_number\_535SRS148511\_contig\_number\_4166SRS148784\_contig\_number\_21391SRS148817\_contig\_number\_2434SRS148874\_contig\_number\_6510SRS893259\_contig\_number\_2838SRS893285\_contig\_number\_2054SRS893288\_contig\_number\_2894SRS893383\_contig\_number\_36052SRS971275\_contig\_number\_37932


SRS017103\_contig\_number\_35632
species
1
469591


SRS023526\_contig\_number\_11177
species
1
46503

815
family
8

8
genus
816

1
species

SRS144603\_contig\_number\_10800
204516

1
species

SRS104693\_contig\_number\_10191
338188


SRS077335\_contig\_number\_298
species
1
1638779

821

SRS1041095\_contig\_number\_665SRS147377\_contig\_number\_2906
species
2

817
species

SRS142599\_contig\_number\_7748
1

species

SRS046712\_contig\_number\_104
1
818

46506
1
species

SRS015960\_contig\_number\_5133
